# Whole-Genome Sequences of 12 Clinical Strains of Listeria monocytogenes

**DOI:** 10.1128/genomeA.01203-14

**Published:** 2015-02-26

**Authors:** Guojie Cao, Jianmin Zhang, Xuebin Xu, Huiming Jin, Xiaowei Yang, Haijian Pan, Marc Allard, Eric Brown, Jianghong Meng

**Affiliations:** a Department of Food Science & Engineering, Shanghai Jiao Tong University, Shanghai, China; b Department of Nutrition & Food Science and Joint Institute for Food Safety & Applied Nutrition, University of Maryland, College Park, Maryland, USA; c Shanghai Municipal Center for Disease Control & Prevention, Shanghai, China; d Division of Microbiology, Center for Food Safety & Applied Nutrition, Food and Drug Administration, College Park, Maryland, USA

## Abstract

Listeria monocytogenes is a foodborne pathogen of global concern due to the high mortality rate among immunocompromised patients. Whole-genome sequences of 12 strains of L. monocytogenes from humans were reported. The availability of these genomes should provide useful information on the evolutionary history and genetic diversity of L. monocytogenes.

## GENOME ANNOUNCEMENT

Listeria monocytogenes is a Gram-positive intracellular pathogen that causes severe disease among immunocompromised individuals (1). L. monocytogenes is ubiquitous and able to survive in a wide range of environmental conditions (2). Most human listeriosis has been attributed to serotypes 1/2a, 1/2 b, and 4 b (3). In China, serotypes 1/2a, 1/2 b, and 1/2c account for over 90% of the L. monocytogenes strains found in food (4). Whole-genome sequencing has become an essential tool for investigating the evolution, diversity, and pathogenicity of foodborne bacterial pathogens (5–9). The draft genomes of 12 L. monocytogenes strains from hospitalized patients in Shanghai, China, between 2007 and 2012 were reported.

Genomic DNA was isolated from overnight cultures and sequenced using MiSeq (Illumina, San Diego, CA) to obtain draft genomes. Genomic data were assembled using Newbler 2.6 (Roche, Branford, CT), including SHL001 (33 contigs, 2.95 Mb, 476,844 bp *N*_50_ contig size, and 2,964 identified genes), SHL002 (22 contigs, 3.12 Mb, 301,779 bp *N*_50_ contig size, and 3,113 identified genes), SHL004 (18 contigs, 3.01 Mb, 579,300 bp *N*_50_ contig size, and 3,018 identified genes), SHL005 (17 contigs, 2.88 Mb, 437,049 bp *N*_50_ contig size, and 2,859 identified genes), SHL006 (30 contigs, 2.93 Mb, 476,139 bp *N*_50_ contig size, and 2,959 identified genes), SHL007 (43 contigs, 2.98 Mb, 355,359 bp *N*_50_ contig size, and 2,995 identified genes), SHL008 (26 contigs, 3.01 Mb, 293,078 bp *N*_50_ contig size, and 2,990 identified genes), SHL009 (32 contigs, 2.87 Mb, 541,739 bp *N*_50_ contig size, and 2,866 identified genes), SHL010 (84 contigs, 3.08 Mb, 259,950 bp *N*_50_ contig size, and 3,112 identified genes), SHL011 (17contigs, 2.87 Mb, 543,519 bp *N*_50_ contig size, and 2,847 identified genes), SHL012 (23 contigs, 2.93 Mb, 355,398 bp *N*_50_ contig size, and 2,906 identified genes), and SHL013 (20 contigs, 2.86 Mb, 358,858 bp *N*_50_ contig size, and 2,806 identified genes). These sequences were annotated using the NCBI Prokaryotic Genomes Automatic Annotation Pipeline (10).

### Nucleotide sequence accession numbers.

The draft genome sequences of these 12 L. monocytogenes strains are available in GenBank under the accession numbers APIB00000000, APIC00000000, APID00000000, APIE00000000, APIF00000000, APIG00000000, APIH00000000, APII00000000, APIJ00000000, APIK00000000, APIL00000000, and APIM00000000.
